# Psychometrics Anonymous: Does a Transparent Data Sharing Policy Affect Data Collection?

**DOI:** 10.5334/pb.503

**Published:** 2019-09-19

**Authors:** Julia Charlotte Eberlen, Emmanuel Nicaise, Sarah Leveaux, Youri Léon Mora, Olivier Klein

**Affiliations:** 1 Université Libre de Bruxelles (ULB), BE; 2 Université Lumière Lyon 2, FR

**Keywords:** privacy, anonymity, GDPR, psychology, methods, data accessibility, open science

## Abstract

As researchers, we are advised to share our data to improve transparency and increase the reproducibility of experiments. Simultaneously, making data freely accessible can raise ethical questions regarding the participants’ privacy. We first outline the challenges regarding “open data” for researchers in light of the GDPR. Then, we turn to the impact of an open-access data sharing policy on the participants: could the participants’ knowledge about the future use of the data alter the data itself? Through two pre-registered studies (N = 193, collected on campus and N = 543, online participation), we investigate whether disclosing that anonymized data will be publicly shared vs. not shared influences a potential participants’ intention to take part in the study. Using both frequentist and Bayesian analysis, we conclude towards an absence of effect of a difference in data sharing policy on scores in the Big Five questionnaire and social desirability, careless response behavior, and results in the anchoring paradigm. In the second study, a lexicometric analysis of participants’ reactions to openly sharing data reveals a readiness to share data and support transparency under the condition of preserved anonymity. Hence, if anonymity can be ensured, there seems to be no methodological or ethical drawback in transparent and open data sharing policies for many psychological studies.

## Introduction

In today’s digital world, personal information is both harder to protect than ever before and holds an increasing monetary value to companies in the business of buying online advertisements (Pachilakis, Papadopoulos, Laoutaris, Markatos, & Kourtellis, 2019). Google, Facebook and other companies possess large quantities of user data. But psychological research is also increasingly data rich, with larger sample sizes, data downloaded from social media platforms, and studies including a multitude of variables. In parallel, the credibility revolution (Vazire, 2018) calls for more transparency, including making datasets openly accessible on online platforms such as the open science framework (OSF, osf.io) and the open science data cloud (intended for large datasets, https://www.opensciencedatacloud.org/). In addition, with the introduction of the General Data Protection Regulation (GDPR, *Regulation (EU) 2016/679 of the European Parliament and of the Council of 27 April 2016 on the protection of natural persons with regard to the processing of personal data and on the free movement of such data, and repealing Directive 95/46/EC (General Data Protection Regulation)*, 2016) in the European Union (EU), researchers have to abide by rules on how to handle personal and sensitive information. Given this climate of increased data availability and uncertainty in how data is actually treated “behind the scenes”, it is ethically crucial (and, depending on local regulations, often compulsory) to inform potential participants that their anonymous data will be accessible to others before they can proceed the study. Many practical challenges are addressed in an invited forum titled “Challenges in Making Data available” (Simons, 2018). But what if the very information that data is made publicly accessible, has an influence on willingness to participate, or on participants’ behavior during the study itself?

### Modern challenges in data collection

The transparency revolution leads to more researchers striving to make data openly accessible to other researchers specifically and the interested public in general. The aforementioned OSF is only one tool to share raw study data. Many other research data repositories exist and can be explored (e.g., re3data.org by the non-profit organization DataCite; databrary.org by Databrary, 2012). This provides the opportunity to increase the credibility of scientific findings, and enable and facilitate new insights. For academic research, largely funded by public money, it is a welcome development that the resulting data is more and more available to the public. But under which circumstances can untreated research data from psychological studies actually be shared?

Until the introduction of the GDPR in May 2018, there was no uniform regulation on how data relating to citizens and inhabitants of the EU were to be treated, and what rights regarding personal data these individuals have. Since then, European citizens and residents are more in control of what we share, and how the data is treated. Now, companies collecting and handling personal data give us the theoretical ability to consent to the data usage.

But regardless of the EU’s effort to restrict the abuse of personal data, the past and present behavior of both industrial and research data collectors have not inspired trust in users. The persistent but unfounded rumor that Instagram is collecting audio data to improve ad targeting based on overheard conversations (Facebook Newsroom, 2016; Goldman, Vogt, Pinnamaneni, Bennin, & Marchetti, 2017) can be seen as an example of how past “bending the rules” behavior and data breaches influence user trust. In light of legal, illegal and rumored exploitations of voluntarily and involuntarily shared personal data, it seems almost ridiculous to make data sharing and protection in scientific research the center of an ethical discussion. After all, even in large-scale studies, the number of participants and variables is typically not anywhere near what social media companies have at their disposal, and data collection is typically anonymous, or at least anonymized.

However, consider social psychology and its sketchy history of handling data: Some of the most famous studies have not considered privacy or even consent at all. This was the case of Henle and Hubble, who hid under the beds of college students as to better observe natural behavior (1938). It can be argued that consent alone is not sufficient to guarantee an ethically sound study, with Milgram’s obedience studies (1963) and the Stanford Prison experiment (Haney, Banks, & Zimbardo, 1973) providing anecdotal support. Even though these studies have long since been criticized and were the very reason to introduce ethics committees in psychological research, we have since developed new issues surrounding informed consent and data protection. One such example is the ongoing scandal around (former) researchers from Cambridge University involved in Cambridge Analytica (Wong, 2019): An institution which should inspire trust, and be a mark of high research standards, saw its name associated with massive data misuse, and data obtained without consent (although here, the University itself bears no responsibility).

This is a case of extreme misconduct. But in our daily practice, researchers in psychology do not necessarily work to inspire trust within our study subjects either. We very rarely disclose the research question in advance, thereby taking away the “informed” part of the consent. In many cases, and for many participants, this practice is fairly benign and has no serious consequences. However, we cannot tell whether the individual’s decision to participate or not in our study would have been different if they were in full knowledge of its purpose. Chances are, they would have thought it too boring for their time.

More importantly for the context of this work, truly anonymizing a dataset is more complex than simply removing name, age, and location. We can safely assume that every single one of our participants has some form of online presence. This means that for every person there is a dataset to cross-reference with ours, thereby increasing the potential for re-identification. Just recently, researchers demonstrated how little data is necessary to re-identify up to 99.98% of all individuals (Rocher, Hendrickx, & Montjoye, 2019).

While this might not necessarily be problematic for typical participants in datasets without any sensitive content, it can take only one or two unusual data points to make a participant stand out. In a typical psychological experiment, the most common combination of features is first year psychology student, 18–25 years of age, and female. Being a male student aged between 30–40 reduces the list of candidates to just a few participants. To identify the male participants by name, it takes just little additional information: roughly the year in which the study was conducted, and, for example the corresponding cohort’s Facebook group, or an automated search on twitter to find profiles that match the description (male, “psychology” mentioned in profile, location corresponding to that of the university…). Again, for most studies, the additional information gained from crossing several datasets is benign. But would all participants who are unaware of the exact hypothesis of the study, and of the way their data could be re-identified still agree to participate? In addition to the recombination of openly accessible data, there is the possibility that data is leaked, servers hacked, or data integrity otherwise compromised without the researcher’s (or participant’s) consent. Although this has – to our knowledge – not happened in psychology yet, there has been a case where the personal data from users of a website facilitating extramarital affairs, leaked after a hack, was combined with (in the US) publicly available voter registration information (Arfer & Jones, 2019). Although the researchers did not themselves steal the data, the website users were likely not aware of this risk when first signing up.

Data collected within, from citizens, or residents of the EU, need to be compliant with the GDPR, thereby avoiding some of the extreme consequences legally permissible in other parts of the world. Of course, anonymous and fully anonymized data, meaning data that are no longer identifiable based on “available technology at the time of processing and technological development” are excluded from the regulation (article 26, GDPR). Consequently, a good open access dataset is one where personal data has never been asked in the first place. In all other cases, data needs to be anonymized. However, if personal data is indispensable for a study, participants need to be informed of all potential use cases of their data. Making participant data accessible without restriction implies that the researcher cannot (and should not) control by whom, when and why a given dataset is reused, which makes this restriction rather complicated. We might use a science-minded data repository, but the people downloading the freely accessible data are not necessarily researchers (although see Databrary, 2012, for an exception, more can be found at re3data.org).

Even if the data can be anonymized, it is not trivial to understand when a complex dataset is actually not at risk of re-identification. As stated above, it can be surprisingly easy to identify outliers in an anonymous dataset. But given enough knowledge and resources, sometimes even entire anonymized datasets can be de-anonymized with legal means. This was demonstrated by Narayanan and Shmatikov (2006) for the famous Netflix dataset, by de Montjoye, Hidalgo, Verleysen, and Blondel (2013) for cellphone mobility data such as the publicly available data from sites like Foursquare, and most recently as a general case by Rocher, Hendrickx, and Montjoye, (2019).

But even for datasets containing personal information with the participants’ consent, there is a major caveat with its own set of problems: The GDPR grants participants who provide personal data the right to withdraw their consent to use the data. First, it is entirely unrealistic that the researchers can fulfill their responsibility to find all potential copies. Second, this poses questions regarding consequences of withdrawing individual data points, and how to handle subsequent changes in results and conclusions in scientific publications. Here, the only solution is to increase trust and decrease the risk of later data withdrawal requests by providing participants with honest consent forms, including a statement on data sharing. However, such a statement has the potential to influence the response behavior of the study subjects.

### Study rationale

Given the concerns detailed above, we aimed to investigate the possible impact of data sharing policies on study participation. If making anonymous data publicly available is a cause for concern for some participants, we should expect a higher rate of refusal to participate if the study consent contains an open access data policy disclaimer. We planned to investigate this question using a Chi-square analysis. To continue this line of thought, it is possible that people who chose to participate in the study despite knowing that their anonymous data will be openly accessible have different personality characteristics like high scores in openness to experiences on the Big Five Questionnaire. This may limit the external validity of the study due to participants’ self-selection (Carnahan & McFarland, 2007). Conversely, knowing that individual data will be shared can lead participants to change their behavior throughout a study more towards what is deemed socially acceptable compared to participants whose data will only be shared as aggregate statistics. In addition, knowing that data will be shared can induce participants to read and respond more carefully than if they know that their answers will be hidden in the group response. Finally, we wondered whether the findings of well-established psychological study paradigms would be influenced by participants’ knowledge about the subsequent use of their data: is it possible that there will be a change in typical response patterns? We conducted the following studies with these practical questions in mind and chose participant populations typically used for psychological studies to provide an ecological context. Due to this concern for practical validity, we also opted for the more frequently used questionnaire when more than one tool was available and did not exclude participants based on their correct recollection of the data sharing policy.

The rationale for the analyses of these secondary dependent variables was as follows: We conducted frequentist and Bayesian ANOVAs and t-tests to establish whether we can reasonably conclude that there is a difference in mean responses between the two consent conditions in personality, social desirability, and the anchoring paradigm. To assess whether the scale reliability would differ between condition, we compared Cronbach’s alpha for each subscale with frequentist statistics. We compared homogeneity of variances using Levene’s test to find out whether there is a difference in spread of participant response, indicating more error variance in one over the other case. This rationale also applies to the Chi-square test and the corresponding Bayesian contingency table test on the careless response items: is there one condition where more participants answer without paying attention the question content?

All materials and analysis scripts are available for inspection and reuse. We can only provide files for the participants in the “open access” consent condition for both studies conducted. To provide as much detail as possible without compromising the consent for those participants who were in the “no sharing” condition, we created detailed metadata with the R codebook package (Arslan, 2019). All files are available on this project’s osf.io page https://osf.io/zyux4/.

## Study 1 – On-Campus, Off-line Participation

### Methods

The first study was conducted on campus, with volunteer participants in the form of a paper-and-pencil questionnaire.

### Participants

Sample size was determined beforehand based on practical implications: 9 students participating in a research seminar collected participant data until each student had obtained at least 20 participants. Of the potential participants, one person refused to take part after having read the consent, and one abandoned the questionnaire before completion. Both were included in the main analysis, for a total N = 193, with 111 participants reporting being women, 81 men, with one missing value. We excluded 1 participant from all further analyses based on the person’s score outside 3 absolute deviations around the median, a procedure robust with regards to sample size and the values of the outliers themselves (MAD, Leys, Ley, Klein, Bernard, & Licata, 2013). In the anchoring paradigm, we excluded additional participants based on the same procedure to avoid confounds due mistakes in the unit (i.e., when participants indicated 20,000 instead of 0.02 million).

### Material

The study material consisted in booklets containing one of two different versions of a consent form and the questionnaires described below. Regarding the main independent variable, the two consent forms varied only in one sentence: In the non-sharing consent, we noted that “My identity will be treated anonymously, and I won’t be identifiable from my answers. My anonymous data won’t be accessible to thirds.” In the sharing condition, we noted that “My identity will be treated anonymously, and I won’t be identifiable from my answers. The data files will be accessible to thirds online, via the public website osf.io, so that other researchers can analyze the data.” Of note, the latter is a longer, more complicated phrasing than the first. The consent was followed by 3 questionnaires in counterbalanced order. The 39-item French validation (Plaisant, Courtois, Réveillère, Mendelsohn, & John, 2010) of the Big Five questionnaire (Costa & McCrae, 1999) is a frequently used questionnaire designed to capture five major dimensions of a person’s personality, referred to as openness to experience, conscientiousness, extraversion, agreeableness and neuroticism. We use it to measure whether the different data sharing policies have an impact on the expression of personality on the one hand, and on the reliability and variance of participant responses on the other.

The second measure was the 18-item sub-scale “other-deception” of the French social desirability scale by Tournois, Mesnil and Kop (2000). This sub-scale was created based on translations of several frequently used social desirability questionnaires including the social desirability scale (Crowne & Marlowe, 1960) used in study 2.

We chose three items of the anchoring paradigm (Jacowitz & Kahneman, 1995) as used in Klein and colleagues (2014) to assess whether the data sharing policy would in any way impact response behavior in a well-established finding. In this task, participants are asked to estimate a number (in our case: the height of Mt. Everest, the number of inhabitants of Chicago, and the number of babies born each day in the US). Critically, there are two between-participant conditions: one group receives an underestimation, while a second sees an overestimation as reference points. Typically, this leads to group differences in mean estimations of the target number (R. A. Klein et al., 2014). We chose the specific questions based on the magnitude of their effect size (R. A. Klein et al., 2014) and presumed applicability in a Belgian context. Here, the participants were further divided into two subgroups per consent condition, resulting in four different groups (data shared/low anchor, data shared/high anchor, confidential data/low anchor, confidential data/high anchor).

Finally, 7 careless-response items, based on the bogus items in Meade and Craig (2012) were mixed into the two questionnaires.^1^ The items were translated and adapted to the context in the study. We did not intent to exclude participants based on their incorrect responses to these items. Rather, we included them as a measure of participation quality: would participants read and respond more carefully in one consent condition than in the other?

Participant attribution to both consent and anchoring condition was randomized: The questionnaires were assembled including a cover page, with consent condition marked on the last page, and given in random order to the students administering them. Therefore, unless a participant had a question while reading the consent, the administering student was unaware of the study condition.

### Procedure

Data collection was done at highly populated places on the main campus of the Université Libre de Bruxelles, mainly during lunch hour. The questionnaire was presented as a “personality questionnaire”. Experimenters followed a predetermined script and strict instructions not to test people known to them personally. Participants were encouraged to carefully read the instructions and inform the experimenter once they had finished reading. Once participants had signed the consent, experimenters removed both the cover page and the signed consent from the questionnaire. This was done to increase participants’ trust in data anonymity while assuring experimenter’s blindness to the consent condition. Completed questionnaires were stored separately from the consent. Questionnaires from people who read the consent but did not agree to continue were marked with a completion code and stored with the other consent forms. At the end of the questionnaire administration, participants were thanked, debriefed and provided with a link to our osf.io page.

### Data analysis

We used R (Version 3.5.2; R Core Team, 2018)^2^ for all our analyses. As we had only one refusal to participate we deviated from our pre-registered analysis protocol.

Specifically, given the low expected values in the “refusal” cells of the table, we could not use a Chi-square test. Instead, we analyzed whether the difference in participation is within the error margins of measurement error when comparing two samples Klein (2014).

### Results

There was only one person who declined to participate (consent condition: open access to data). This corresponds to a measurement error of 2%, with an upper limit of a 95% confidence interval of 3.14%, for an acceptable difference of measurement error of 5% between the conditions).

**Figure 1:**
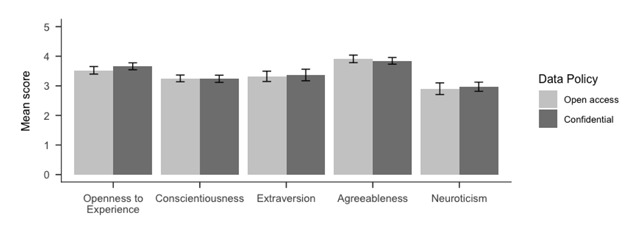
Mean scores with 95% confidence intervals by subscale of the French Big Five personality questionnaire (Plaisant et al., 2010). Answers given on 5-point Likert scales.

Although there was only one refusal to participate, we still tested as planned for a systematic difference between the two groups’ average responses. We used a mixed measures 5 (within: Big Five subscales) by 2 (between: consent condition) ANOVA. For all Bayesian analyses throughout the two studies, we used the priors provided by the R package BayesFactor (Version 0.9.12.4.2; Morey & Rouder, 2018). We identified a main effect of subscales *F*(2.95,559.7) = 45.56, *MSE* = 0.72, *p* < .001, \hat \eta _G^2 = .162, BF_10_ = 1.93 × 10^32^, but moderate support for the absence of a main effect of consent condition *F*(1,190) = 0.29, *MSE* = 0.50, *p* = .589, \hat \eta _G^2 = .000, BF_10_ = 0.08 (see Figure 1). There was strong support for the absence of an interaction consent and Big-Five subscales: *F*(2.95,559.7) = 0.34, *MSE* = 0.72, *p* =.790, \hat \eta _G^2 = .001, BF_10_ = 0.01. To test whether participant responses were more reliable in one condition than in the other, we compared the Cronbach alphas obtained for each subscale using the method developed and implemented in the R package cocron by Diedenhofen and Musch (2016). We did not find any difference for 4 out of the 5 scales (Extraversion: χ^2^(1) = 0.89, *p* = .34; Agreeableness: χ^2^(1) = 0.01, *p* = .93; Conscientiousness: χ^2^(1) = 0.43, *p* = .51; Neuroticism: χ^2^(1) = 0.08, *p* = .77). For the subscale “Openness to experience”, the results suggest a difference, but only at the alpha level of 0.05, uncorrected for multiple testing, χ^2^(1) = 4.30, *p* = .038, with a higher alpha coefficient for the sharing condition (Cronbach’s alpha = 0.79, CI = [0.72, 0.85]) than for the non-sharing condition (Cronbach’s alpha = 0.66, CI = [0.55, 0.75]).

To assess the impact of the consent on the tendency to respond in a socially desirable way, we conducted a two-sample t-test, Δ*M* = 0.01, 95% CI [–0.18, 0.15], *t*(183.72) = –0.16, *p* = .87, *M* = –0.01 95% HDI [–0.17, 0.16], BF_10_ = 0.16: The frequentist t-test does not allow to reject the null hypothesis of no difference between means, and the Bayesian analysis supports moderately an absence of effect. The Cronbach’s alphas are 0.78, CI = [0.71, 0.84] and 0.86, CI = [0.81, 0.90] for the data sharing and non-sharing condition, respectively, χ^2^(1) = 3.71, *p* = .054). The variance in both conditions is unequal, as indicated by Levene’s test of equality of variances tested at the mean, *F*(1,190) = 6.15, *p* = .014.

The number of participants who failed two or more of the seven attention checks within the questionnaire was statistically not different, χ^2^(1) = 2.32, *p* = .128 95% CI [–0.03, 0.27]. However, the Bayesian contingency analysis suggests no support for the null hypothesis of no difference between conditions, BF_10_ = 0.70.

For the three anchoring paradigm questions, we conducted all analyses. First, we analyzed the effect for the number of babies born each day in the US (low anchor: 100, high anchor: 50,000). We included only complete responses and answers within 3 absolute deviations from the median, which lead to an N = 165 (Leys et al., 2013). In the conducted 2 × 2 between participant ANOVA, we found a main effect of anchoring condition, *F*(1,161) = 44.06, *MSE* = 829,634,592.57, *p* < .001, \hat \eta _G^2 = .215, BF_10_ = 2.64 × 10^7^, but neither of the consent condition, *F*(1,161) = 0.02, *MSE* = 829,634,592.57, *p* = .89, \hat \eta _G^2 = .000, BF_10_ = 0.19 (moderate support for an absence of effect), nor an interaction between consent and anchoring conditions, *F*(1,161) = 0.94, *MSE* = 829,634,592.57, *p* = .33, \hat \eta _G^2 = .006, BF_10_ = 0.35 (anecdotal support for an absence of effect). The distribution of values within the low and high anchoring Levene’s test for equality of variances was comparable between the two consent conditions for the low anchoring value, *F*(1,80) = 0.39, *p* = .53, and the high-anchoring value *F*(1,81) = 0.00, *p* = .98. For the estimation of the height of Mount Everest (low anchor = 600 m, high anchor = 13,800 m), there remained N = 174 participants (Leys et al., 2013). As for the previous question, we found a main effect for low vs. high anchoring in a 2 (low anchoring vs. high anchoring) by 2 (consent: sharing vs. no sharing of data) ANOVA, *F*(1,170) = 99.86, *MSE* = 13,709,665.03, *p* < .001, \hat \eta _G^2 = .370, BF_10_ = 8.84 × 10^15^, but moderate support for an absence of effect *F*(1,170) = 0.18, *MSE* = 13,709,665.03, *p* = .67, \hat \eta _G^2 = .001, BF_10_ = 0.16 nor for the interaction between consent and anchoring condition, *F*(1,170) = 0.18, *MSE* = 13,709,665.03, *p* = .67, \hat \eta _G^2 = .001, BF_10_ = 0.24. As before, there was a comparable distribution of values around the mean for both consent conditions in the low and high anchoring condition, respectively (*F*(1,83) = 0.35, *p* = .55, *F*(1,87) = 0.01, *p* = .93). Finally, for the question on the number of inhabitants of Chicago (low anchor = 0.2 million, high anchor = 5 million), we conducted the same tests as above on N = 165 participants and found a main effect of anchoring, *F*(1,161) = 33.46, *MSE* = 8.09, *p* < .001, \hat \eta _G^2 = .172, BF_10_ = 4.47 × 10^5^ but moderate support for the absence of effect, *F*(1,161) = 0.15, *MSE* = 8.09, *p* = .7, \hat \eta _G^2 = .001, BF_10_ = 0.22 and moderate support for an absence of interaction effect between consent and anchoring, *F*(1,161) = 0.01, *MSE* = 8.09, *p* = .91, \hat \eta _G^2 = .000, BF_10_ = 0.23. Equal distribution of values around the mean for the consent conditions in the low- and high-anchoring condition, respectively was confirmed, *F*(1,81) = 0.13, *p* = .717, *F*(1,80) = 0.42, *p* = .52. For all three anchoring questions, the effect size was situated much closer to the results of the original study than to the Many Labs replication (2014) (Babies: *d_Original_* = 0.93, *d_Privacy_* = 1.04, *d_ManyLabs_* = 2.42, Mount Everest: *d_Original_* = 0.93, *d_Privacy_* = 1.54, *d_ManyLabs_* = 2.23; Chicago: *d_Original_* = 0.93, *d_Privacy_* = .91, *d_ManyLabs_* = 1.79).

### Discussion

Our study was aimed to investigate whether people would more often refuse to participate if their anonymous data would be shared publicly online than when it remained confidential. This hypothesis was not confirmed. Although the person who did not participate explicitly cited the data sharing as the reason for their refusal to participate, this remains anecdotal evidence at this point. Concerning the secondary tests whether there is a difference in the data obtained under different disclosed data sharing policies, the results suggest that there is largely no difference between the two groups. We therefore conclude that including a data sharing statement in the consent is unlikely to impact the data quality in a study in these well-established, frequently used measurements. The small difference identified between the Alpha Cronbach values for the subscale “openness to experience” of the Big Five vaguely resembles traditional significance criteria. But given the number of statistical tests conducted, this is more likely due to multiple testing at the alpha level of 0.05 rather than a true difference between groups. The “other-deception” sub-scale of the French social desirability scale (Tournois & al., 2000) seems to indicate more variability between the two conditions. This difference is not found in the scale mean, but the Cronbach’s alpha and the difference in variability. Given that measures of social desirability are known to be difficult to design and formalize, and the limited sample size of this study, we are hesitant to interpret these differences as relevant for practical purposes. An exploratory repetition of the analyses on a sample including only participants who remembered the data sharing policy in their consent confirms this interpretation (see Supplementary material/osf.io for the additional results).

## Study 2 – Online Participation

Sassenberg and Ditrich conducted a formal study of the use of online platforms for data collection and found a considerable increase in the last 10 years (2019). Therefore, we conducted a second version of this study using this popular, convenient, and fast data collection tool. Again, our main question was whether people based their decision to participate on the data sharing conditions announced in the study consent. As participants in online studies take part for monetary compensation, it could be argued that the motivation to participate is even less influenced by the data sharing conditions. However, we can also make the argument that people place value not only on their time, but also on the control over their data. Therefore, people might decide against participation, or modify their behavior when their anonymous data is shared. In an exploratory aim, we also analyzed whether participants in a paid context read the consent at all (see SoM). After all, many of the contributors on participant platforms are familiar with the typical content of consent forms. We emphasized the phrase on sharing (or not) anonymous data as much as possible. If, despite this emphasis, people participate and do not modify their behavior, it can safely be argued that making data publicly available has no impact on participation. We also included an open question to find out more about participants’ opinion about open data directly. This was also done in a study by Bottesini and Vazire (2019), who used the Amazon Mechanical Turk platform (MTurk) to recruit presumably American participants. Participants in this study were overwhelmingly in favor of data sharing. Conversely, in the (American) medical domain, Hull et al., (2008) found that participants, although generally in favor of additional research conducted on their samples, wanted to know about this further use of their data. Considering the higher demand for protection of personal data in the EU we wonder whether the opinions mirror the American context.

### Methods

The second study was a variation of the first study, differing in the participant population, recruited via an online platform, and using English versions of the questionnaires (detailed below).

### Participants

Participants were recruited on the online platform Prolific and compensated with £1 for their 10-minute participation. We preselected users to be residents of the EU, English speakers, aged 18 or above, and with an approval score of 80 or above. The last 3 criteria were included to recreate the conditions of a typical online experiment. We determined the sample size based on the planned analysis of the main dependent variable, study participation. For a Chi-square goodness of fit test with a small to medium effect size of w = 0.174, power = 0.95 (df = 1), the g*power software estimated a sample size of N = 584. Although this number includes both complete and abandoned participation, we collected 600 responses to allow for some incomplete cases. However, despite precautions on Prolific, some participants accessed the questionnaire multiple times. We excluded all second occurrences of a participant id as well as those who did not complete the questionnaire or scored above or below 3 MADs in the TIPI (Gosling, Rentfrow, & Swann, 2003) or the social desirability questionnaire (Crowne & Marlowe, 1960), leading to N = 543. Most were aged 21–30 years (N = 288). There were 245 women, 285 men, 4 non-binary participants, and 9 who did not report their gender. In one anchoring paradigm question (number of inhabitants of Chicago), multiple participants had failed to use “million” as the unit of their estimation. We changed the unit if the answer exceeded 100. To avoid a further confound between the anchoring effect and choosing the wrong unit, we excluded data points for each anchoring question based on 3 MADs specifically, but included the respective participant data in all other analyses (see results section for specific N per question).

### Material

Study 2 consisted in two variations of a consent form similar to study 1. We took care to formulate the consent variations in sentences with similar length and highlighting (Non-sharing condition: “Data files will NOT be accessible online to others. My data will only be shared in the form of aggregated statistics, such as mean values based on all study participants”; sharing condition: “Data files will be accessible online to others via a public website osf.io so that other researchers will be able to reanalyze the data collected.”). Participants were required to consent (or not) via two radio buttons. We used the Ten Item Personality Inventory (TIPI, Gosling et al., 2003). This short version of the Big Five personality questionnaire (Costa & McCrae, 1999) is reduced to two items for each dimension, one of them reverse-coded. As in the long version, the dimensions are openness to experience, conscientiousness, extraversion, agreeableness, and neuroticism. We furthermore chose the most frequently used social desirability scale (Crowne & Marlowe, 1960), and the same three items of the anchoring paradigm as used in (Klein et al., 2013) and careless response items based on Meade and Craig (2012) as in study 1. To assess whether participants remembered the data sharing condition, we asked them to answer four comprehension questions on the different parts of the consent, with one item relating to the data sharing policy. As a further modification, we included one open response question: “What are your thoughts on open access to participant data in the scientific field?”. All analyses regarding these two items are exploratory. The study was created using the open source software Limesurvey (Schmitz, 2012).

### Procedure

The study was presented on Prolific as a personality test. We assigned participants randomly to one of the two consent conditions and either the low or high anchoring condition, for a total of four different groups (data shared/low anchor, data shared/high anchor, confidential data/low anchor, confidential data/high anchor). Provided people agreed to participate, they then proceeded to the first question on the consent form before completing the questionnaires in randomized order. At the end, participants had the option to fill in the open entry and demographic questions, were debriefed and paid.

### Data analysis

In addition to the software and packages listed in study 1, we used IRaMuTeQ (Loubère & Ratinaud, 2014; Ratinaud, 2009) for the lexical analysis (see https://osf.io/zyux4/ for scripts and data treatment).

### Results

#### Primary analysis

To test whether participants recruited on an online platform would more often disagree to a study consent stating that anonymous data will be shared publicly as opposed to a closed data policy, we had planned to conduct a Chi-square test. This was not possible as we had only one participant (consent condition: open access) who chose to disagree with the consent. A test of whether this is within the margins of measurement error revealed that the result of the difference of participation in the sharing vs. non-sharing condition is 0.37%, which corresponds to an error margin of 0.72%, CI [–0.35, 1.09]. Due to the nature of the TIPI (Gosling et al., 2003), there is only limited value to using Cronbach’s alpha as an indicator of scale reliability. Consequently, the values are very low, ranging between 0.029 (Agreeableness) and 0.74 (Extraversion). Despite this, they are consistent between the two study conditions (Extraversion: χ^2^(1) = 0.04, *p* = .849; Conscientiousness: χ^2^(1) = 2.06, *p* = .15; Neuroticism: χ^2^(1) = 0.01, *p* = .93; Openness to experience: χ^2^(1) = 0.01, *p* = .92. Values could not be computed for the Agreeableness subscale due to negative Cronbach’s alpha in one condition). The item correlations, by subscale and consent condition, are displayed in Table 1.

**Table 1:** Inter-item correlations of the TIPI by subscale and consent condition.

	Extraversion	Agreeableness	Conscientiousness	Neuroticism	Openness to Experience

Open access	0.59	–0.07	0.40	0.55	0.33
confidential	0.58	0.08	0.26	0.55	0.32

*Note*: Correlations are calculated after recoding of reverse-coded items.

**Figure 2:**
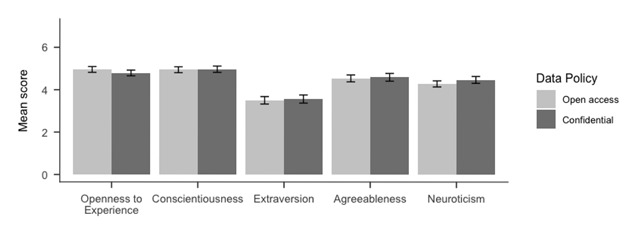
Mean scores of the Ten-Item Personality Inventory (Gosling et al., 2003) by subscale and consent condition. Answers were given on 7-point Likert scales.

We conducted a mixed ANOVA with the TIPI scale as within, and consent condition as between participant factor. Although there was a strongly supported main effect of TIPI subscales, *F*(3.72,2014.05) = 144.67, *MSE* = 1.46, *p* < .001, \hat \eta _G^2 = .152, BF_10_ = 3.75 × 10^93^, we found no effect of consent *F*(1,541) = 0.28, *MSE* = 2.65, *p* = .59, \hat \eta _G^2 = .000, and strong support for this absence of effect, BF_10_ = 0.05 as well as very strong evidence for an absence of interaction effect between consent and the TIPI dimensions *F*(3.72,2014.05) = 0.50, *MSE* = 1.46, *p* = .722, \hat \eta _G^2 = .001, BF_10_ = 0.00, see Figure 2.

We had used the Marlowe-Crowne Social Desirability Questionnaire (Crowne & Marlowe, 1960) to investigate whether there would be a difference between the two data sharing conditions. Concerning the reliability of the social desirability questionnaire, we found moderate support for an absence of difference in scale means between conditions, *t*(504.75) = 0.51, *p* = .61 *M* = 0.01 95% HDI [–0.02, 0.03], BF_10_ = 0.11. Cronbach’s alpha was comparable between the two conditions (χ^2^(1) = 1.74, *p* = .187). Levene’s test for equality of variances likewise indicates no difference in response distribution. We conducted a Chi-square test based on the number of participants who failed to respond correctly to two or more of the careless response items, χ^2^(1) = 0.00, *p* = > .99 95% CI [–0.17, 0.17].

For the anchoring paradigm, we conducted a two (anchoring: low vs. high) by two (consent: data is shared vs. not shared) between-participant ANOVA and a Levene’s test for equality of variances on all three questions. For the question on the number of babies born each day (N = 450), we found the predicted main effect of anchoring *F*(1,446) = 197.74, *MSE* = 172,366,187.67, *p* < .001, \hat \eta _G^2 = .307, BF_10_ = 4.86 × 10^33^ but moderate evidence for the absence of effect of consent *F*(1,446) = 0.29, *MSE* = 172,366,187.67, *p* = .59, \hat \eta _G^2 = .001, BF_10_ = 0.11. We did not observe an interaction between consent and anchoring *F*(1,446) = 2.45, *MSE* = 172,366,187.67, *p* = .12, \hat \eta _G^2 = .005 BF_10_ = 0.43 (anecdotal evidence for the absence of effect). The variances across both consent conditions are comparable (*F*(1,204) = 0.28, *p* = .59 and *F*(1,242) = 0.94, *p* = .333, respectively). For the anchoring question on the height of Mount Everest (N = 400), we found similar results: The ANOVA indicated a main effect of the anchoring condition, *F*(1,396) = 77.77, *MSE* = 3,233,573.71, *p* < .001, \hat \eta _G^2 = .164, BF_10_ = 2.93 × 10^14^, no main effect for consent, *F*(1,396) = 0.81, *MSE* = 3,233,573.71, *p* = .36, \hat \eta _G^2 = .002, BF_10_ = 0.29, and no interaction between consent and anchoring condition, *F*(1,396) = 0.20, *MSE* = 3,233,573.71, *p* = .653, \hat \eta _G^2 = .001, BF_10_ = 0.17. The homogeneity of variances was not influenced by data sharing, neither in the low (*F*(1,226) = 1.64, *p* = .2), nor in the high-anchoring condition (*F*(1,170) = 2.18, *p* = .14). Concerning the anchoring paradigm question on the number of inhabitants of Chicago (N = 494), the results were similar. One more time, we found the main effect of anchoring, *F*(1,490) = 63.22, *MSE* = 3.70, *p* < .001, \hat \eta _G^2 = .114, BF_10_ = 6.36 × 10^11^, but not of consent, *F*(1,490) = 0.08, *MSE* = 3.70, *p* = .77, \hat \eta _G^2 = .000, BF_10_ = 0.10 or the interaction of consent and anchoring condition, *F*(1,490) = 0.00, *MSE* = 3.70, *p* = .98, \hat \eta _G^2 = .000, BF_10_ = 0.14. Like before, Levene’s test did not indicate a difference in variance for the two consent variations in either low and high anchoring condition: *F*(1,253) = 0.49, *p* = .48 and *F*(1,237) = 0.00, *p* = .98. The effect size of the “Baby” anchoring question was situated between the Cohen’s d of the original study (Jacowitz & Kahneman, 1995) and the ManyLabs replication (R. A. Klein et al., 2014), *d_Original_* = 0.93, *d_Privacy_* = 1.33, *d_ManyLabs_* = 2.42. However, the effect sizes of the other two anchoring questions were lower than in the original study (Mount Everest: *d_Original_* = 0.93, *d_Privacy_* = 0.88, *d_ManyLabs_* = 2.23; Chicago: *d_Original_* = 0.93, *d_Privacy_* = .71, *d_ManyLabs_* = 1.79).

#### Exploratory analysis

One additional question to ask is whether participants actually remember the content of the consent, specifically, do they remember if their data will be shared? We found that out of 542 participants, 301 correctly remember their sharing condition. Across the two conditions, however, there was a considerable difference: a Chi-square test and corresponding Bayes factor indicate that participants in the non-sharing condition are much more likely to remember this information, χ^2^(1,*n* = 542) = 58.43, *p* < .001, BF_10_ = 1.99 × 10^12^. We repeated all analyses on the reduced dataset, which did not lead to any considerable changes (See supplementary material/osf.io for details).

For the exploratory analysis of thoughts on open access to participant data, we pre-processed all written responses provided by the participants and then subjected this corpus to a treatment by hand coding and two different lexicometric analyses (for details on the preparation, please see https://osf.io/zyux4/). Our final corpus includes 505 texts, consisting of a total of 8359 words. Table 2 sums up the main lexical characteristics of this corpus.

**Table 2:** Main lexicometric characteristics of the free entry corpus.

N° of texts	N° of words	Average n° of words per text	N° of forms	Total n° of hapaxes	Percentage of hapaxes in corpus

505.00	8,359.00	16.55	949.00	470.00	5.62

*Note*: One text = one participant contribution, Form = word reduced to its word stem, e.g. ‘support’ for ‘supported’ and ‘supporting’, Hapax = word with single occurrence.

We first hand coded the answers to the open question on a scale from one (data should not be shared) to five (data can be shared without any restrictions). Coders were blind to each other’s ratings, but not to consent condition. Not all participants answered this question, and not all answers explicitly addressed the participant’s position towards data sharing, for a total of N = 423 participants providing an answer (N = 232 in the sharing condition). The classification of answers correlated to a satisfying degree between two judges (*r* = .85, 95% CI [.78, .89], *t*(103) = 16.17, *p* < .001), with an overlap of 78.1%. The participants generally expressed their support and consent to data sharing, and to similar degrees in both consent conditions, both according to a classic t-test Δ*M* = –0.15, 95% CI [–0.05, 0.35], *t*(380.26) = 1.50, *p* = .134 and a Bayesian estimate *M* = 0.15 95% HDI [–0.04, 0.35], BF_10_ = 0.33.

**Figure 3:**
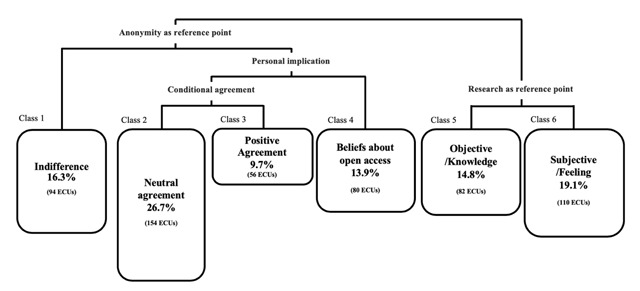
Dendrogram of Reinert analysis. 6 classes were identified automatically and named by hand (see text for details). ECU = Elementary Context Unit.

In a second step, we conducted two lexicometric analyses on the corpus: a hierarchical descending classification analysis (HDC, also known as Reinert analysis), and an analysis of lexical correspondences, conducted on forms occurring more than 7 times in the corpus. These two types of analyses were used for their complementarity (Loubère, 2016). The HDC analysis allowed us to highlight lexical worlds in the form of dominant themes and lexical groups in our corpus of texts. This was done by identifying the vocabulary that significantly characterizes each class (Reinert, 1983), using the R-based software IRamuteq (Ratinaud, 2009). This classification distributes the statements into different classes which can be distinguished by the contrast of their vocabulary (Kalampalikis, 2005). The second method is based on co-occurrence of words and makes it possible to highlight the links existing between the different concepts provided by the participants. This analysis is conducted based on the context of a given word and by graphically presenting the existing associations and links between the words in the corpus. The HDC analysis enabled us to extract six stable classes integrating 100% of the elementary context units (ECUs) of the corpus. The dendrogram (Figure 3) summarizes the classification and representativeness of each class in relation to the entire corpus. We attributed class and node names after examining the vocabulary of the different classes. On this dendrogram we see a clear distinction of two types of arguments provided by the participants. On the one hand, “research” itself was mentioned as a topic. Participants stated their knowledge about open access in the scientific field and their feelings about the usefulness or the way open access works (classes 5 and 6).

**Figure 4:**
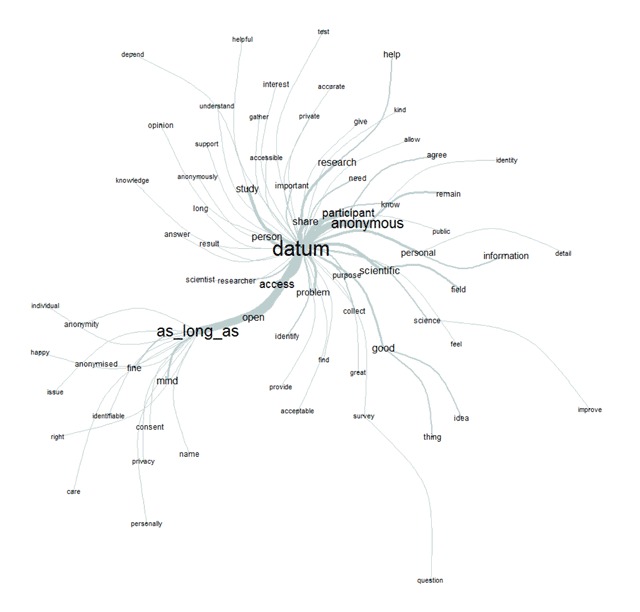
Graphical representation of the analysis of similarities by co-occurrence for the text corpus (see text for details).

On the other hand, participants referenced the importance and centrality of anonymity (classes 1 to 4). From the co-occurrence analysis (Figure 4), we can distinctly see the relevance of anonymity in participants’ answers. One of the most frequently mentioned and most strongly related words to the notion of data is “anonymous”. The second is the expression: “as long as” which stands for the conditional aspect of open access data. This condition is also linked to “anonymity”, “privacy” or “consent”. If the condition (of anonymity) is met, then the participants seem to be “fine” and “happy” with the idea, or at least they don’t “mind” or “care”.

We found the same idea of conditional agreement in the HDC analysis. Indeed, in the answers given by the participants, we can observe how participants seem to relate to the issue of open access. The notion of a “neutral agreement”, meaning a general but not enthusiastic support (class 2) is very present. Indeed, roughly a quarter (26.7%) of the corpus’ content is related to this second class, making it the biggest class of the corpus. Some participants mention a “positive agreement” (class 3) or on an “indifference” (class 1) on the other side of the spectrum. However, throughout these positions, the question of anonymity remains essential.

### Discussion

In the second study, we were interested in extending the findings of study 1 to the context of an online platform with EU participants. We had predicted that more participants would abandon a study if the consent indicated that their anonymous data would be shared online, compared to a condition where data would remain confidential. Only one participant overall chose to disagree. A second erroneously disagreed, only to re-do the study choosing to agree to the consent. Given this anecdotal evidence of measurement error, we cannot reliably infer that the single participation refusal is linked to the consent form itself rather than an unintentional “I disagree” mouse click. Our hypothesis of a difference in overall participation was therefore not confirmed. In addition, we found no evidence of a difference between the two experimental conditions, neither with regards to Big Five scores, nor the tendency to respond in a socially desirable way, nor in any measurement of data quality we applied to our data.

Participants were generally in favor, or at least not opposed, to share their data. However, we found a considerable amount of concern for anonymity, and a wish to be informed about the researcher’s intention to share their data. It is interesting to contrast this agreement to data sharing amongst participants with the reported *actual* practices and attitudes of sharing study data amongst psychologists (Houtkoop et al., 2018), which can be euphemistically described as hesitant. It seems like the participants, the backbone of our research, can also serve as an example with regards to data sharing.

## General Discussion

The purpose of this project was to provide insight into whether or not disclosing open access to anonymous participant data would influence either participation itself, or the quality of the data obtained in a psychological study. We investigated this question with two different participant samples typically used in psychological research: a first group recruited on campus, and a second group solicited via an online platform. Neither study indicated any difference in participation between the data sharing and non-data-sharing consent condition.

Although the first study, conducted with a sample of students recruited off-line provided some weak evidence for small differences in data quality, these differences are likely to be due to a relatively small sample size. The second, larger study provides support for this explanation, as there is no evidence for even a small bias due to different data sharing policies.

We conclude from this that at least within the limits of data collected for research purposes, there lies no harm, and much benefit, in disclosing the data sharing policy explicitly in the consent form. Most notably, the only participant who purposely refused to continue after having read the consent referred to the open access data sharing policy as their reason for refusal. Although this preference seems to be rare, we consider it worthwhile and a long-term investment in a positive relationship between the general public and the psychological sciences to be open about our intentions. The importance of transparency and the individuals’ control over what they agree to share (or not) is supported by the lexical analysis of the open-response question in study 2. There are almost no “hard” rejections to sharing participant data with other scientists, but an overwhelming majority of participants wishes to remain anonymous. This is especially interesting considering that some participants explicitly mention that they are in favor of data reuse in a scientific, but not a commercial context (e.g., “(…) I disagree if any commercial use comes into play”, “(…). If it’s for research purposes and not marketing then you all have my blessing”). However, if we opt for a truly open access format of data sharing, the control over who actually downloads the data is out of our hands. It is consistent with the wording of our consent forms (“…so that other researchers will be able to reanalyze the data collected.”), a weakness of our study, that participants interpret “open access” to mean “scientific purposes only”.

This positive attitude towards scientific research, as well as the willingness to participate in the first place, can be due to the formulation we used, but also to the way we recruited our participants. In study 1, we relied on a student population at a university. We assume that these participants are unlikely to be science sceptics, and probably have a higher motivation to contribute to a scientific study conducted by peers than the general population. In addition, once they were engaged to participate, it might have been difficult to refuse after having read the consent. For study 2, there might be similar mechanisms at work: Prolific is a website destined for research. The people who sign up to work as participants on this platform are likely to be doubly motivated: First, they know in advance that their data will be used for research purposes, and chose to work on this platform rather than one with a more general design. Second, participants receive a fixed payment for their contribution, so once they have invested the time to click on a task and read the consent, there is an incentive to complete the study. Given this high level of engagement these participant groups are unlikely to refuse to participate, whatever the consent. While this is true, these groups also constitute our discipline’s typical participant pool.

Another concern to researchers conducting psychological studies is the content of our questionnaires: Personality questionnaires, social desirability scales and the anchoring paradigm all are fairly benign. We cannot reasonably claim that the results we found in the context of these two studies can be generalized to other types of research. Possibly, studies conducted on different issues like mental health are affected by a policy of openly sharing anonymous participant data. The case is also clearly different for studies conducted on participants from a specific sub-population, such as patients, members of minorities, minors, or people who are likely to be re-identified because of the nature of the collected data. However, in those cases, the question is much less whether we should fully disclose our policy regarding data sharing, but rather whether we should grant access to the raw data at all. The GDPR does not oblige researchers based in the EU, or conducting studies with EU residents, to disclose all future (research-focused) uses of anonymous datasets, or even whether or not a dataset not containing personal information will be openly shared. Yet, many of the participants in our study felt that researchers should ask permission to share anonymous data before the beginning of the study.

To a certain extent, this desire to be informed is in contrast with the finding that only slightly more than half the participants remembered how their data will be treated. Nonetheless, this leaves two possibilities. First, it is conceivable that the other half didn’t pay more attention because of the announced, fairly generic, topic of “personality research”, as opposed to a more sensitive issue. Providing a data-sharing policy in the consent enables participants to take an informed decision on their participation no matter the research topic. Furthermore, we cannot exclude that disclosing a data-sharing policy was appreciated by those who did remember it. This view is supported by Hull et al., (2008), who state that although participants are generally in favor of reuse of their medical samples, they also wished to be informed and/or asked. Interestingly, they also identified the same support for sample reuse for research but not commercial aims, which, again, cannot be controlled by the original authors once the data is on a public repository.

This brings us to the conclusion that including a statement on data sharing in the consent form, while not mandatory under the GDPR, is ethically advisable in order to maintain a positive long-term relationship with those who make our research possible. Overall, our results indicate that participants are willing to contribute not only to the advancement of science, but also to a better, more transparent and responsible research culture. We can – and should – see this as a sign that there is (still) trust in the scientific process, despite the ongoing replication crisis, and keep up the work to earn this trust.

## Footnotes

Due to an error in the material, 8 participants filled out a questionnaire with a repetition of one of the careless-response items instead of 7 different items.

We, furthermore, used the R-packages *afex* (Version 0.23.0; Singmann, Bolker, Westfall, & Aust, 2019), *BayesFactor* (Version 0.9.12.4.2; Morey & Rouder, 2018), *car* (Version 3.0.2; Fox & Weisberg, 2011), *cocron* (Version 1.0.1; Diedenhofen, 2016), *dplyr* (Version 0.8.0.1; Wickham, François, Henry, & Müller, 2019), *ggplot2* (Version 3.1.1; Wickham, 2016), *here* (Version 0.1; Müller, 2017), *pacman* (Version 0.5.1; Rinker & Kurkiewicz, 2018), *papaja* (Version 0.1.0.9709; Aust & Barth, 2018), *psych* (Version 1.8.12; Revelle, 2018), and *tidyr* (Version 0.8.3; Wickham & Henry, 2018).
